# Spatial averaging method based on adaptive weight for imaging photoplethysmography

**DOI:** 10.1117/1.JBO.28.8.085003

**Published:** 2023-08-30

**Authors:** JongSong Ryu, HyonSam Ryu, Shili Liang, SunChol Hong, Yueqi Lian, Zong Zheng

**Affiliations:** a Northeast Normal University, School of Physics, Changchun, China; b University of Science, Faculty of Physics, Pyongyang, Democratic People’s Republic of Korea; c State Academy of Sciences, Institute of Mechanical Engineering, Pyongyang, Democratic People’s Republic of Korea; d Academy of Ultramodern Science, Kim Il Sung University, Pyongyang, Democratic People’s Republic of Korea

**Keywords:** spatial averaging, adaptive weight, imaging photoplethysmography, sub-region of interest, signal-to-noise ratio

## Abstract

**Significance:**

Imaging photoplethysmography (iPPG) is a non-contact measuring technology for several physiological parameters reflecting personal health status without a special sensor. However, the pulse signal obtained using the iPPG usually is contaminated by various noises, and the intensity of the interesting pulse signal is relatively weak compared to the noises, emphasizing the necessity of obtaining high-quality pulse signals to measure physiological parameters correctly.

**Aim:**

Various regions of the face harbor distinct pulse information. We propose a spatial averaging method based on adaptive weights, which can obtain high-quality pulse signals by applying different weights to facial sub-regions of interest (sub-ROIs; sROIs).

**Approach:**

First, the facial ROI is divided into seven sROIs and the coarse heart rate (HR) is calculated from them. Next, the signal-to-noise ratio (SNR) of the raw signal obtained from each sROI is calculated using the coarse HR, and then a high-quality pulse signal is obtained by assigning positive or negative weights to each sROI based on the SNRs.

**Results:**

We compare our method with others through the quality analysis of the obtained pulse signals using the self-collected database and the public database PURE. The comparison results show that the proposed method can provide a better pulse signal compared to other methods under various resolutions and states.

**Conclusions:**

This proposed method can obtain the pulse signal with better quality, which is helpful to accurately measure physiological parameters, such as HR and HR variability.

## 1 Introduction

The cardiac cycle is the complete cycle of events in the heart from the beginning of one heart beat to the beginning of the next.^1^ Through cardiac cycle analysis, important physiological parameters such as heart rate (HR) and heart rate variability (HRV) can be obtained, which can help predict and diagnose a person’s heart vascular disease.

Currently, electrocardiography (ECG) and photoplethysmography (PPG) are the most common technologies for cardiac cycle analysis. The ECG obtains the electrocardiogram signals by first attaching the electrodes of the sensors to the different parts of the human body and then getting electrical signals from several parts of the human surface. The PPG uses electrooptical technology to obtain a pulse signal by detecting a change in blood volume in the skin tissue through contact between the sensor and the human skin. Although the accuracy of measuring physiological parameters with these two technologies is high, the electrodes or the sensors require direct contact with the human body, which causes inconvenience to people and is unacceptable for special populations like burn-patients, newborn babies, people with sensitive skin, and so on. They are also not suitable for everyday measurements because they use special sensors that are not commonly used in daily life. The imaging PPG (iPPG) is a technology that can analyze the cardiac cycle without contact with the human body, which is developed from the PPG, and the pulse signal is obtained by recording the color change of the skin with a camera using ambient light as a light source. The iPPG can overcome the limitations of ECG or PPG since it measures physiological parameters using a camera, a ubiquitous device in our everyday lives, instead of special sensors requiring contact with the human skin.

Since the change in skin color caused by heartbeat is very small, the pulse signal obtained by iPPG is very weak and easily affected by ambient light changes and the relative movement between a camera and people. The measurement of physiological parameters based on iPPG can be divided into three steps: the first is to obtain the pulse signal from the video by image processing technology; the second is to remove the noise in the pulse signal by some signal processing techniques; and the third is to measure the physiological parameters. The quality of the pulse signal obtained in the first step often affects the measurement accuracy of physiological parameters, especially if noise dominates. Therefore, it can be said that the step of obtaining the pulse signal is the most basic and important.

The quality of the pulse signal depends on the selection of the ROI and the spatial averaging method. In the measurement of physiological parameters based on iPPG, any exposed skin region can be used as the ROI. In some studies,^2^^–^^12^ the lower leg, palm, and forearm were used as the ROI, but most studies used the whole face or parts of it, which was not easily covered and well perfused. The studies using a face region as the ROI can be divided into two categories: one is using the rectangular region surrounding the whole face or a predefined percentage of it as the ROI;^13^^–^^17^ the other is using a certain region of the face as the ROI.^18^^–^^29^ The comparative analysis of the pulse signals obtained from the forearm (dorsal), forearm (ventral), forehead, palm, hand (dorsal), cheek, nose, and whole face was performed, and the results showed that the forehead and cheek can provide the high-quality pulse signal.^18^^–^^20^ On the other hand, some authors use facial skin region as ROI^21^^–^^27^ or adaptively select ROI providing high-quality pulse signal.^28^ The disadvantage of the above studies^21^^–^^28^ is that they use a specific region of the face as the ROI and obtain the pulse signal from it, so it cannot effectively utilize the pulse information of different parts in the face. In Ref. 19, distinct weights were assigned to each small block to effectively utilize the pulse information from various facial regions. The input images were divided into small blocks, and the raw signals were obtained from the small blocks. The coarse HR was calculated to apply the same weights to the raw signals obtained from the small blocks. On the other hand, the ratios between the area under the power spectral density of the raw signals obtained from the small blocks within a specific region around the coarse HR and the area outside of that region were calculated. Then, a threshold was established during setting the weights. If the calculated ratio of a small block was found to be lower than the threshold, the weight assigned to that small block was set to 0. Conversely, if the calculated ratio exceeded the threshold, the weight was set to the calculated ratio itself. Finally, the weights were applied to the raw signals obtained from the small blocks to extract high quality pulse signal. However, this method neglects regions that contain less pulse information, limiting its ability to fully utilize the pulse information within the ROI. Furthermore, since the coarse HR was calculated using the raw g-channel, it is susceptible to larger errors when the noise component outweighs the pulse component, especially during subject movements. Consequently, this can lead to difficulties in obtaining accurate weights for subsequent analysis.

In our previous paper,^29^ the facial ROI was divided into seven sub-regions of interest (sROIs) considering the distribution of blood vessels, skin thickness and skin surface temperature, and suitable fixed weights (FWs) were determined for each sROI through experiments conducted on a database. Among these FWs, the two sROIs with the lowest signal-to-noise ratio (SNR) were assigned values less than or equal to 0, whereas the remaining sROIs were assigned positive values. By utilizing fixed positive or negative weights, this method effectively leveraged the sROIs that contained less pulse information, all while requiring small computation. However, since the distribution of pulse information varies among individuals, the spatial averaging method based on FWs may have limitations in terms of adaptability. On the other hand, in our previous paper,^30^ we proposed a modified plane-orthogonal-to-skin based method (POS) for motion-robust HR measurement. Since the modified POS enhanced the performance of POS by projecting onto the CbCr-plane in YCbCr color space, we refer to this modified POS as POS(CbCr) throughout the paper. In this paper, we propose a novel spatial averaging method that employs adaptive weights for seven sROIs to enhance the quality of pulse signals. We improve the accuracy of the weights by calculating the coarse HR using POS(CbCr). Furthermore, by assigning adaptive positive or negative weights to the seven sROIs, we not only effectively utilize sROIs with less pulse information but also improve the method’s adaptability. The self-collected database and the public database PURE^14^ are used to verify the performance of the proposed method.

## 2 Methods

### 2.1 ROI Selection

**Fig. 1:**
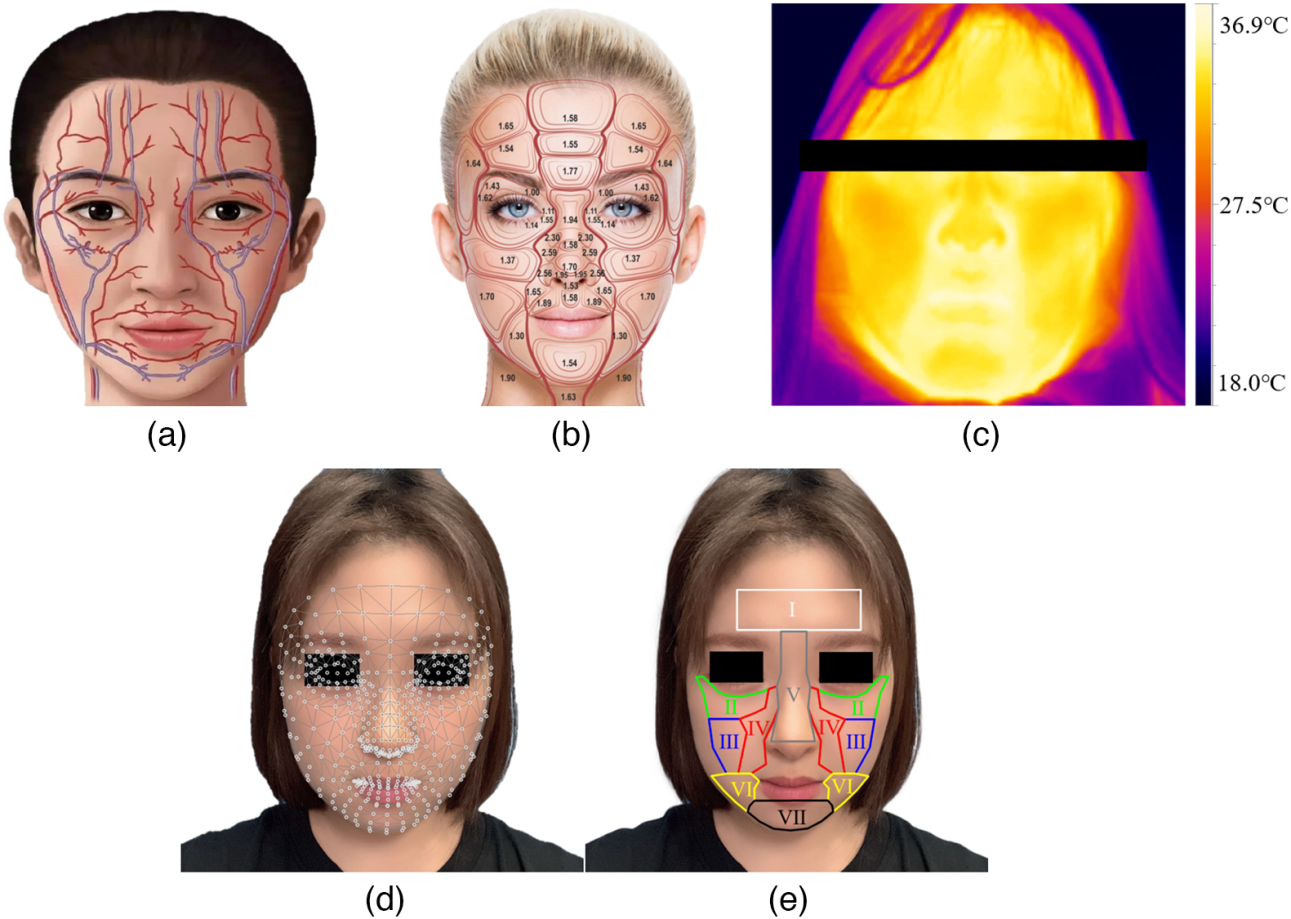
(a) The distribution of blood vessels, (b) anterior view of epidermal relative thickness values.^31^ (c) Skin surface temperature, (d) detected facial landmarks, and (e) selected sROIs.

As the heart expands and contracts, there are quasi-periodic changes in the amount of hemoglobin in the capillaries of the dermis, which change the color of the skin. The iPPG-based methods to measure the physiological parameters use a camera to record such skin color changes to obtain the pulse signal. Therefore, the measurement of physiological parameters based on iPPG will be affected by the skin thickness and the distribution of blood vessels in the ROI. In addition, the quality of the pulse signal obtained by iPPG is also related to the area of ROI. On the other hand, as shown in Fig. 1, the distribution of blood vessels, skin thickness, and skin surface temperature in different parts of the face are different. In this paper, the facial ROI is divided into seven sROIs considering the distribution of blood vessels, skin thickness, skin surface temperature, and the area of sROIs on the face.

In this paper, the facial sROIs are detected and tracked using MediaPipe Face Mesh.^32^ MediaPipe Face Mesh utilizes machine learning techniques to infer 3D surface geometry, enabling precise estimation of 468 3D facial landmarks. Notably, it achieves the detection of facial landmarks by leveraging only a single camera input, eliminating the need for a specialized depth sensor. Due to the lightweight model architecture of the solution, the detection speed is also very fast. The sequence number of facial landmarks used to detect seven sROIs is shown in Table 1.

**Table 1:** The sequence number of facial landmarks used to detect sROIs.

sROI	Sequence number of facial landmarks
I	67, 297, 334, 105
II	111, 143, 35, 31, 228, 229, 230, 231, 232, 233, 47, 100, 101, 117, 340, 372, 265, 261, 448, 449, 450, 451, 452, 453, 277, 329, 330, 346
III	214, 212, 36, 101, 346, 411, 434, 432, 266, 330
IV	245, 233, 47, 100, 101, 36, 212, 186, 165, 102, 198, 174, 465, 453, 277, 329, 330, 266, 432, 410, 391, 331, 420, 399
V	193, 417, 465, 399, 344, 115, 174, 245
VI	214, 212, 186, 61, 43, 204, 211, 170, 169, 135, 138, 434, 432, 410, 291, 273, 424, 431, 395, 394, 364, 367
VII	204, 211, 170, 140, 171, 175, 396, 369, 395, 431, 424

### 2.2 Coarse HR Estimation

To calculate the SNRs of the raw signals obtained from the sROIs, the HR needs to be known. In Ref. 19, the raw g-channel signals obtained from the sROIs were added together to obtain a coarse pulse signal and then a coarse pulse signal in the time domain was converted into the frequency domain, and finally, the peak in the frequency domain was taken as the coarse HR. However, along with the movement of subjects, there will be noises with high energy in the coarse pulse signal, so the error between the coarse HR and the ground truth HR will be increase, and finally the appropriate weights cannot be obtained. In this paper, the limitation of Ref. 19 is overcome by utilizing POS(CbCr) with noise removal capability to calculate the coarse HR.

First, seven raw signals sic^(t) are obtained from the selected seven sROIs using spatial averaging

sic^(t)=∑x,y∈ΩisROIPic(x,y,t)|ΩisROI|,c∈{r,g,b}, (1)

where c represents the channel of the frame, $P_{i}^{c}(x,y,t)$ is the pixel value at the position (x,y) of c channel at time t in the $i^{\prime }$th sROI, $\Omega_{i}^{\mathrm{sROI}}$ is the area of the $i^{\prime }$th sROI, and sic^(t) represents the raw signal of c channel for $i^{\prime }$th sROI.

Next, the raw signals obtained from the sROIs are added together to obtain the coarse pulse signal of the c channel sc^(t), as follows:

sc^(t)=∑i=17sic^(t). (2)

And then, POS(CbCr) is applied to sc^(t) to obtain a noise-removed coarse pulse signal p(t). POS(CbCr) comprises three steps: temporal normalization, projection, and alpha-tuning. The temporal normalization can be accomplished using the following equation:

Xnc(t)=sc^(t)μ(sc^(t)), (3)

where $X_{n}^{c}(t)$ represents temporally normalized signal of the c-channel and μ(·) denotes the average operator that calculates the average value. The projection step involves projecting the temporally normalized RGB signals onto the CbCr-plane in the YCbCr color space, which is expressed by the following equation:

$$S(t)=U\cdot X_{n}^{c}(t),$$ (4)

where $U=((-0.168,-0.331,0.499{)}^{T},(0.499,-0.418,-0.081{)}^{T}{)}^{T}$ is a projection matrix and $S(t)=(s_{p}(t)$, $s_{m}(t){)}^{T}\in R^{2\times N}$ (N being the total number of frames) represents the result of projecting $X_{n}^{c}(t)$ onto the CbCr-plane. The noise removed coarse pulse signal p(t) is obtained through α-tuning, as depicted in the following equation:

$$p(t)=s_{p}(t)+\alpha s_{m}(t)\;\text{with}\;\alpha =\frac{\sigma (s_{p}(t))}{\sigma (s_{m}(t))},$$ (5)

where σ(·) means the standard deviation operator.

Finally, the p(t) in the time domain is transformed into the frequency domain signal by Fourier transform, and the highest peak corresponding frequency $f_{\mathrm{HR}}^{c}$ is found within the range of (0.7 Hz, 4 Hz).

### 2.3 Weighting Scheme

Every part of the face contains a distinct amount of pulse information, so different weights need to be applied to each part.

In this paper, a novel weighting method is proposed, which adaptively assigns positive or negative weights to sROIs using SNR. The coarse HR calculated in Sec. 2.2 is used to calculate SNRs of the raw g-channel signals obtained from each sROI. The equation for calculating SNR of the $i^{\prime }$th sROI (${\mathrm{SNR}}_{i}$) in this paper is as follows:

$${\mathrm{SNR}}_{i}=10\log_{10}(\frac{\overset{4}{\underset{f=0.7}{\sum }}(U(f)h_{i}(f){)}^{2}}{\overset{4}{\underset{f=0.7}{\sum }}((1-U(f))h_{i}(f){)}^{2}}),$$ (6)

where $h_{i}(f)$ represents the spectrum of the input signal (where f denotes frequency) obtained from $i^{\prime }$th sROI and U(f) is a binary template window with two values: 1 and 0. A value of 1 indicates that the frequency falls within two specific windows: one window is near the fundamental frequency of $f_{\mathrm{HR}}^{c}$(i.e., [$f_{\mathrm{HR}}^{c}-0.1$, $f_{\mathrm{HR}}^{c}+0.1$]), whereas the other window is near the first harmonics (i.e., [$2f_{\mathrm{HR}}^{c}-0.2$, $2f_{\mathrm{HR}}^{c}+0.2$]). A value of 0 indicates that the frequency falls outside of these two frequency windows.

The weights used in this paper are calculated as follows:

$$h=\frac{1}{7}\overset{7}{\underset{i=1}{\sum }}{\mathrm{SNR}}_{i}-x_{1}(\frac{1}{7}\overset{7}{\underset{i=1}{\sum }}{\mathrm{SNR}}_{i}-\underset{i}{\min}\;{\mathrm{SNR}}_{i}),$$ (7)

w^i=SNRi−h, (8)

wi={w^i,w^i≥0x2·w^i,w^i<0, (9)

where ${\mathrm{SNR}}_{i}$ is the SNR of the raw g-channel signal obtained from the $i^{\prime }$th sROI [see Eq. (6)]; min() is a minimum operator. In addition, h represents a threshold value used to determine whether the weight should be positive or negative and $w_{i}$ is the weight assigned to the $i^{\prime }$th sROI. In this paper, we assign $x_{1}$ a value of 0.25 and $x_{2}$ a value of 0.2 based on the connection between the variables ($x_{1}$ and $x_{2}$) and SNR discussed in Sec. 4.

Finally, the weights are applied to the raw signals sic^(t) obtained from the sROIs to obtain the pulse signal $s^{c}(t)$, as follows:

sc(t)=∑i=17sic^(t)·wi. (10)

## 3 Materials

### 3.1 Self-Collected Database

Thirty volunteers (17 males and 13 females) aged between 23 and 40 years participated in the data collection. All volunteers certified that they were healthy and received an explanation of the experimental tasks while they signed an informed consent form before starting to collect the data.

During data collection, the volunteers were required to maintain a steady state, and the distance between the volunteers and the camera was about 0.8 m. A webcam (Logitech C922) was used to record the facial videos with a duration of 30 s, a frame rate of 30 fps, and a resolution of 1920×1080 while ground truth pulse signals were recorded using a finger-clip pulse oximeter (Cofoe, Shenzhen, China). The facial skin surface temperature for each volunteer was also recorded using an infrared thermal imager (Micro-Epsilon, Germany). Five sets of data were collected from each volunteer, for a total of 30×5=150 sets of data.

### 3.2 Public Database PURE

A total of 10 volunteers (8 males and 2 females) took part in the data collection under the 6 different states [steady, talking, slow translation, fast translation, small rotation (about 20 deg), and medium rotation (about 35 deg)]. A camera (eco274CVGE) was used to record facial videos with a frame rate of 30 fps and a resolution of 640×480 within about 1 min while the ground truth pulse signals were collected using the finger clip pulse oximeter (pulox CMS50E). The distance between the volunteer and the camera was about 1.1 m.

## 4 Results

In this paper, the quality of the raw signal obtained by the proposed method is evaluated on the self-collected database and the public database PURE, taking the SNR as the evaluation index. The quality of the raw signals obtained from the sROIs selected in this paper is compared. I*, II*, III*, IV*, V*, VI*, and VII* represent the methods used to extract raw signals from their corresponding sROIs. 7-sROI refers to the method described in Sec. 2.2 for obtaining the coarse raw signal. Furthermore, in terms of the quality of the pulse signal, the proposed method is compared with the four methods: the first uses the whole face as the ROI (WH); the second uses the facial skin region as the ROI; the third obtains according to a goodness metric (GM);^19^ and the fourth obtains by adopting the FW.^29^ When the whole face is used to obtain the pulse signal, the region whose width of the detected rectangular face region is reduced by 0.6 times is set as the ROI. When the facial skin region is used to obtain the raw signal, in the region whose width of the detected rectangular face region is reduced by 0.6 times, the skin region detected by human skin color clustering technology^33^ is set as the ROI.

**Fig. 2:**
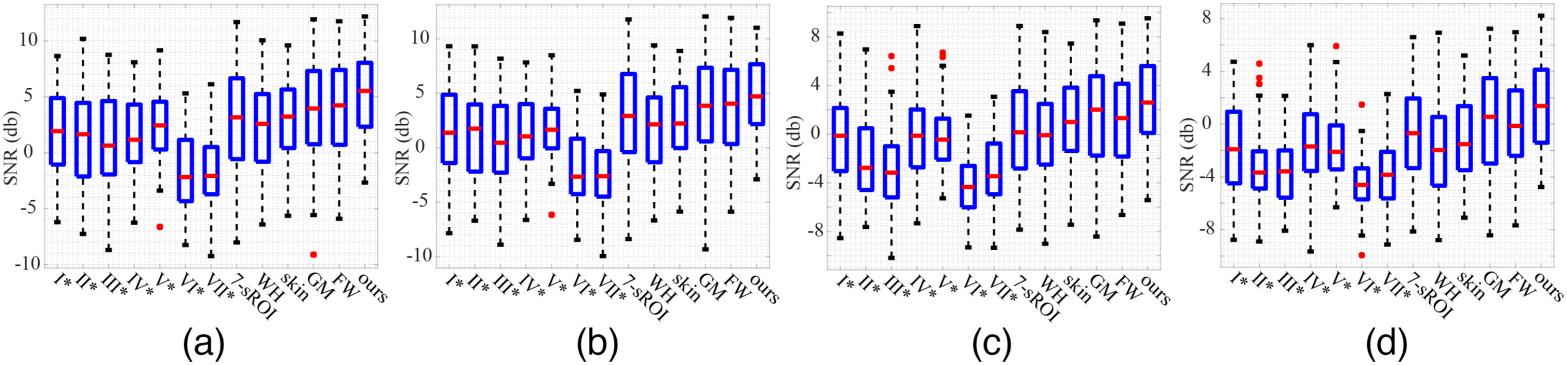
The boxplots of the SNR results of the compared methods for the self-collected database. (a) 1920×1080, (b) 1280×720, (c) 640×480, and (d) 320×240.

Table 2 and Fig. 2 show the SNR results of the signals obtained by different methods for videos with different resolutions in the self-collected database. Here, the videos with resolutions of 1280×720, 640×480, and 320×240 were made by resizing the videos with a resolution of 1920×1080 in the self-collected database. As shown in Table 2 and Fig. 2, for the self-collected database, the proposed method has the best SNR, followed by GM and FW.

**Table 2:** The SNR results of the compared methods on the self-collected database.

	I*	II*	III*	IV*	V*	VI*	VII*	7-sROI	WH	skin	GM	FW	Ours
1920×1080	1.57	1.41	0.94	1.52	2.46	−1.73	−1.86	2.94	2.00	2.92	3.61	3.75	4.97
1280×720	1.47	1.21	0.58	1.29	1.79	−2.03	−2.40	2.79	1.51	2.43	3.51	3.59	4.74
640×480	−0.35	−2.15	−3.03	−0.21	−0.17	−4.18	−3.05	0.25	−0.33	1.02	1.29	1.16	2.68
320×240	−1.96	−3.34	−3.59	−1.60	−1.69	−4.59	−3.79	−0.70	−1.93	−1.07	0.06	0.00	1.21

**Fig. 3:**
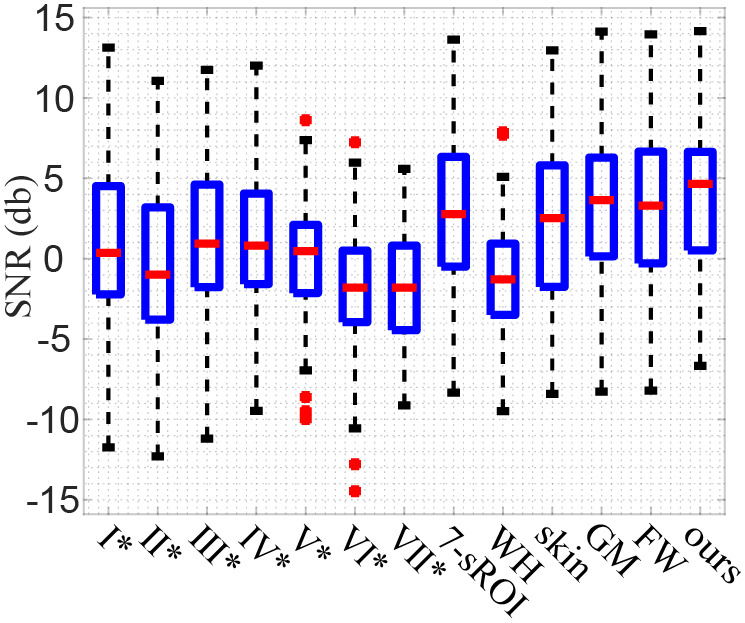
The boxplots of the SNR results of the compared methods for the public database PURE.

The SNR results of the signals obtained by the compared method for the public database PURE are shown in Table 3 and Fig. 3. As shown in Table 3 and Fig. 3, for the public database PURE, as well as the self-collected database, the proposed method also has the best SNR, followed by GM and FW.

**Table 3:** The SNR results of the compared methods for the public database PURE.

I*	II*	III*	IV*	V*	VI*	VII*	7-sROI	WH	skin	GM	FW	ours
0.86	−0.28	1.44	1.26	0.20	−1.95	−1.96	2.79	−1.25	2.18	3.44	3.26	4.03

**Fig. 4:**
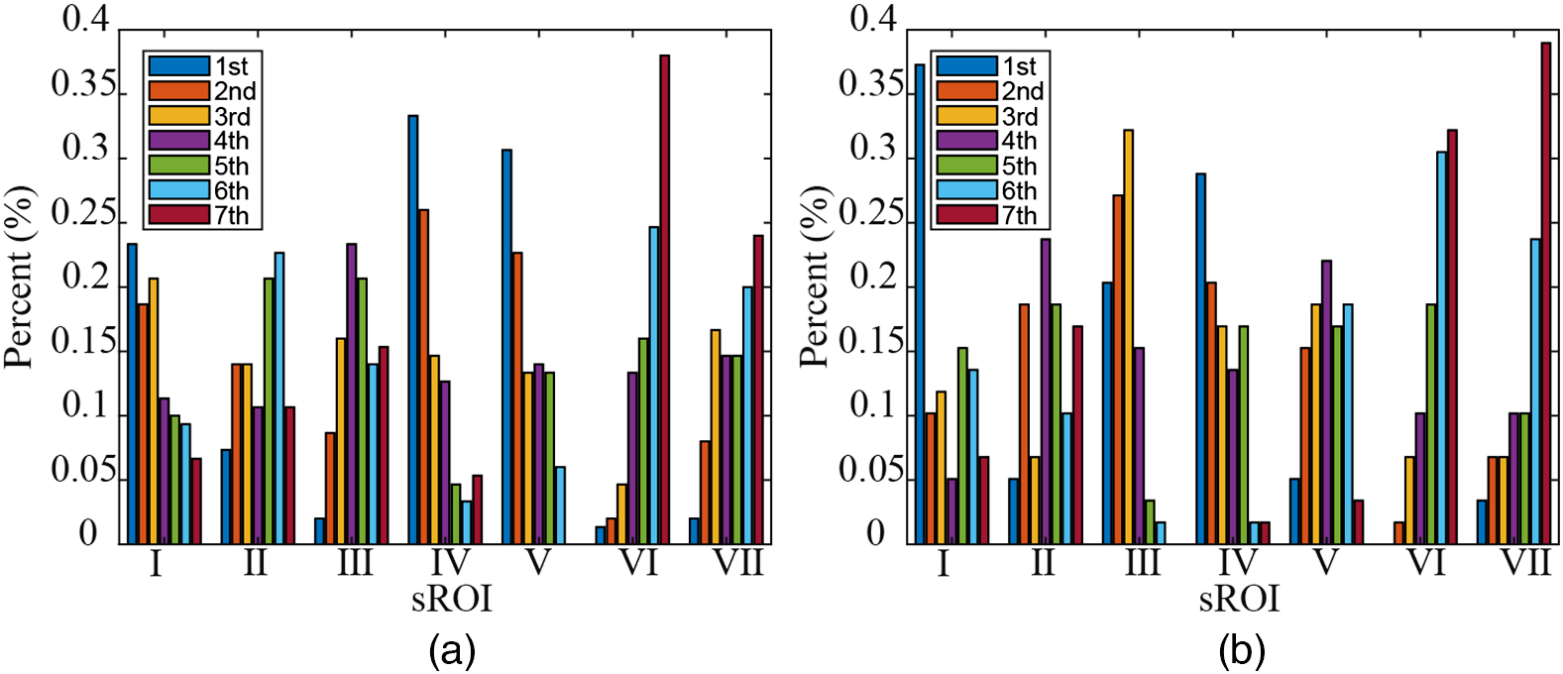
Illustration of the sROI with good SNR varying over different samples. (a) Self-collected database and (b) PURE.

In this paper, a comparative analysis between the qualities of the raw signals obtained from sROIs is conducted. To this end, we first prepare a data matrix with size of M×7 in which M denotes the number of samples and each element denotes the ordinal number of SNR in descending order. Then, we calculate the percentage of each ordinal number in every sROI (i.e., column of the data matrix aforementioned) and show those values for two databases in Figs. 4(a) and 4(b), respectively. For example, “1st” represents the percentage that takes the maximum value in SNRs of the seven SROIs, and “7th” represents the percentage that takes the minimum value. It can be seen from Tables 2 and 3 and Figs. 2–4 that V, IV, and I-sROIs have good SNRs for self-collected database with the resolution of 1920×1080, and IV, III, and I-sROIs have good SNRs for public database PURE.

Table 4 presents a comparison of evaluation metrics for the HR measurement performance of the different methods. The time-domain g-channel signal obtained by the compared method is transformed to the frequency domain by Fourier transform and then the frequency corresponding to the peak is selected from 0.7 to 4 Hz to calculate the HR. Representative statistical metrics, including mean error (ME), mean absolute error (MAE), root mean squared error (RMSE), precision ($P_{\%}$), and Pearson correlation coefficient (r), are utilized to assess the accuracy of HR measurement in comparison to the ground truth HR. The formula for calculating ME, MAE, RMSE, $P_{\%}$, and r is given in Refs. 34 and 35. As indicated in Table 4, the proposed method demonstrates the highest performance for HR measurement.

**Table 4:** Comparison between evaluation metrics of HR measurement performance of the compared methods

		I*	II*	III*	IV*	V*	VI*	VII*	7-sROI	WH	skin	GM	FW	ours
Self-collected database	ME (bpm)	1.03	3.46	2.33	1.15	0.27	6.25	5.34	0.89	1.38	−0.25	0.95	−0.25	−0.10
	MAE (bpm)	2.30	4.51	2.73	1.69	0.99	7.71	6.71	1.45	2.03	0.59	1.38	0.47	0.43
	RMSE (bpm)	7.83	12.57	8.57	6.01	3.70	14.97	14.91	6.46	7.21	1.38	6.43	1.22	1.02
	$P_{\%}$ (%)	92.67	86.67	88.67	94.00	97.33	66.67	78.67	97.33	92.00	98.00	97.33	98.67	100.00
	r	0.75	0.38	0.74	0.86	0.94	0.23	0.27	0.83	0.79	0.99	0.83	0.99	0.99
PURE	ME (bpm)	4.19	6.11	2.47	3.93	5.81	7.00	8.75	1.98	3.61	1.56	1.93	1.82	0.49
	MAE (bpm)	4.79	7.16	3.07	4.56	6.51	8.86	9.28	3.11	7.44	3.60	3.05	2.91	1.47
	RMSE (bpm)	12.52	17.70	11.84	12.75	18.74	18.73	20.66	10.84	17.29	9.80	10.83	10.77	2.23
	$P_{\%}$ (%)	84.21	82.46	91.23	91.23	87.72	64.91	75.44	92.98	75.44	92.98	92.98	94.74	98.25
	r	0.82	0.59	0.82	0.80	0.51	0.55	0.43	0.85	0.59	0.88	0.85	0.85	0.99

**Fig. 5:**
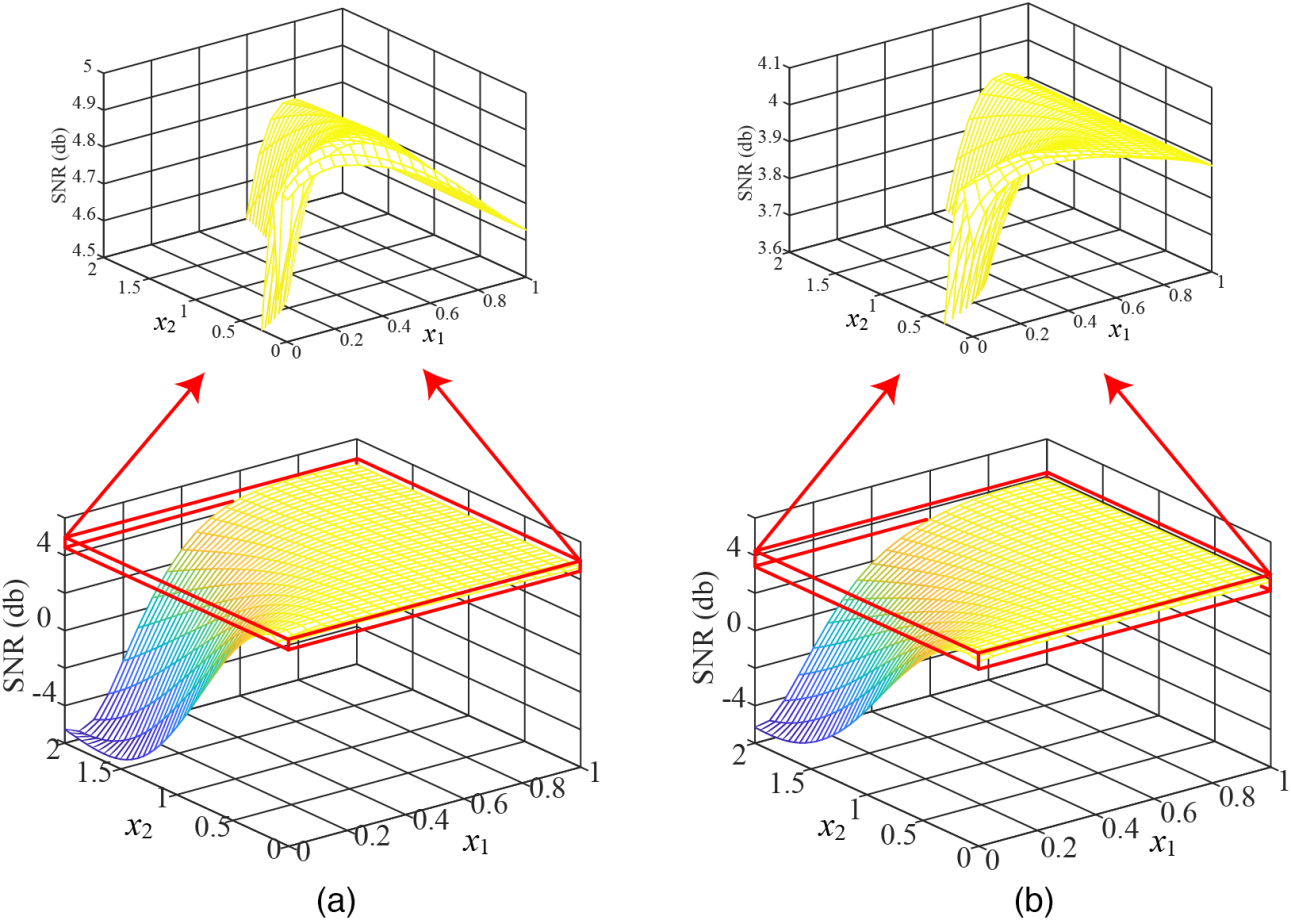
The relationship between the variables and the SNR. (a) Self-collected database and (b) PURE.

Figure 5 shows the relationship between two variables ($x_{1},x_{2}$) and SNR in the proposed method. As shown in Fig. 5, the average SNRs of the signals obtained from the two databases appears to be the maximum value around ($\left(x_{1},x_{2})=(0.25,0.2\right)$, and the farther away from (0.25, 0.2), the smaller the average SNR. And while $x_{1}$ changes, SNR does not change so much. However, for $x_{2}$, we can see a slight change of SNR at (0,0.5) but a significant change at (0.5,2).

**Fig. 6:**
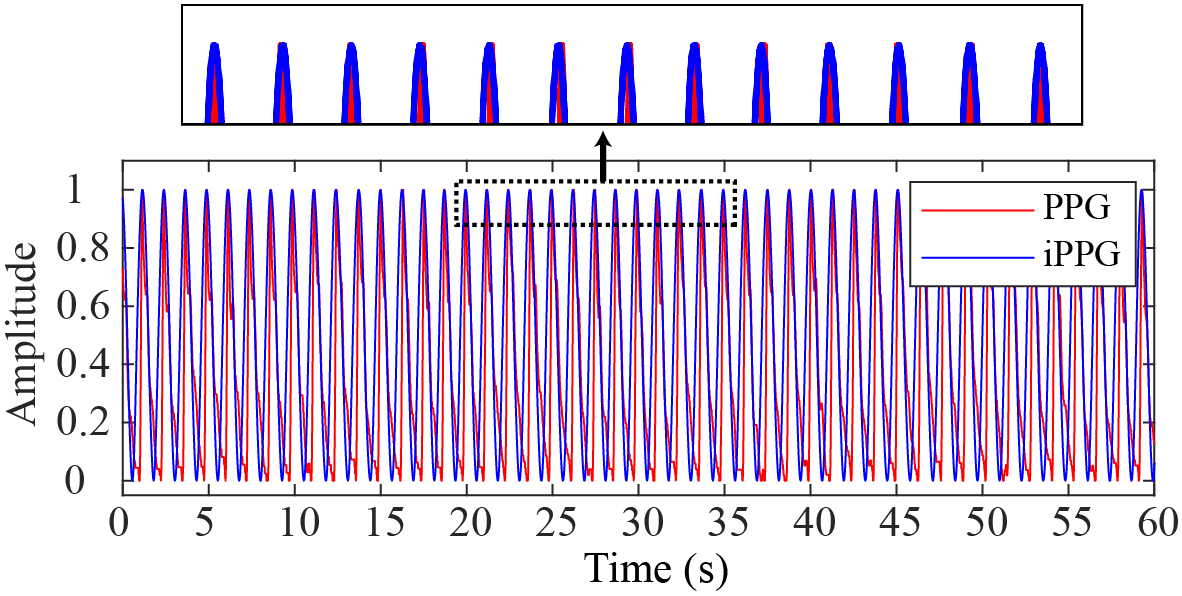
The PPG signal and the iPPG signal obtained through combining the proposed method with SB-CWT(CbCr) for sample data in the medium rotation state from the public database PURE.

In Fig. 6, for sample data in the medium rotation state from the public database PURE, we show the iPPG signal obtained by combining the proposed method and SB-CWT(CbCr),^34^^,^^35^ as well as the PPG signal. It is not difficult to see that the peak positions of the PPG signal and the iPPG signal are almost exactly the same.

## 5 Discussion

Based on the analysis the distribution of facial blood vessels and the SNR of the sROIs, we utilized adaptive weights for the sROIs to conduct spatial averaging, resulting in enhanced raw signal quality during the preprocessing step. The comparison between previous spatial averaging methods based on weights,^19^^,^^29^ spatial averaging methods based on single ROI selection, and the method proposed in this paper demonstrated the superior performance of our method in enhancing the quality of the raw signal, leading to certain improvement in subsequent HR measurement. In Ref. 19, the contribution of the signal with low SNR was mitigated by assigning a small weight, not less than zero, to the sROI exhibiting low SNR. However, in our method, the signal with low SNR was considering as noise, and a negative weight was adaptively assigned to the corresponding sROI. In fact, the phase difference of the pulse signals acquired at any two points within the face is almost zero.^19^ Therefore, assigning a negative weight to the raw signal of the sROI with relatively low SNR could lead to the loss of pulse signal in the resulted raw signal when combined with the raw signals of other sROIs during the weighting process. Alternatively, in most cases, the noises caused by motion and illumination change in the sub-ROIs may exhibit correlation.^36^^,^^37^ Therefore, employing a negative weight for the raw signal of the sROI with relatively low SNR during spatial averaging can achieve a greater reduction in noise compared to the loss of the pulse signal, resulting in an overall improvement in raw signal SNR.

Different methods were also compared at various resolutions of video. As shown in Table 2 and Fig. 2, for all the methods compared, the higher the resolution of the video, the higher the SNR. In addition, although there was a little difference in SNR for the resolution of 1920×1080 and 1280×720, there was a certain degree of difference between 1280×720 and 640×480 and between 640×480 and 320×240. Therefore, considering both computational efficiency and signal quality, the result indicated that the resolution of 1280×720 was the optimal choice for the measurement of physiological parameters based on iPPG under the considered conditions. Moreover, it can be seen that the proposed method can get higher quality raw signals than the compared method at four resolutions.

Our proposed weight-based spatial averaging method adeptly performed simultaneous signal emphasis and noise cancellation, effectively enhancing the quality of the raw signal. Consequently, this method offers the advantage of solely utilizing the facial skin region to reduce noise to some extent, distinguishing it from previous methods^37^^–^^39^ that relied on non-skin regions for noise elimination. Furthermore, significant effort of our study is devoted to enhancing the quality of the raw signals in RGB channels, allowing the proposed method to be freely combined with existing iPPG-based HR and HRV measurement methods. While offering these advantages, the proposed method also exhibits some limitations. The proposed method relies on the coarse HR estimation to determine the weights, so the accuracy of the coarse HR may affect the subsequent measurement of physiological parameters. Indeed, the POS(CbCr) used for coarse HR measurement in this paper was robust to motion and illumination change, but when the intensity of noise is strong, the error of coarse HR measurement may increase. It may also be necessary to redetermine the optimal values of the important factors $x_{1}$ and $x_{2}$ in Eqs. (79). In this study, the comparative optimal ($x_{1},x_{2}$) is determined by employing both the public database PURE and the self-collected database.

To measure HR and HRV more accurately, raw signals of good quality need to be extracted. The proposed method shows better performance than the other compared methods for the self-collected database and the public database PURE. But both databases were collected under conditions where obstacles, such as motion or illumination change, were not severe. Therefore, in order for our proposed spatial averaging method to be generally applied to the preprocessing step of iPPG-based methods, it needs to be fully investigated by more public databases that considered more practical situations. That is, the improvement of the accuracy of the coarse HR estimation and the optimization of the parameter setting for determining the weights should be investigated. We will further study this in the future.

## 6 Conclusion

This paper proposed a method to obtain high-quality pulse signals in which facial ROI was divided into seven sROIs by considering the distribution of blood vessels, skin thickness, and skin surface temperature in the face and used adaptive weights. The proposed method could obtain better quality pulse signals than the existing methods at various resolutions of the videos and under various motion conditions by fusing pulse information from different parts of the face more effectively. The proposed method was able to provide the pulse signal with large SNR, which will be of great help for the easier, more effective, and more accurate measurement of physiological parameters and predict and diagnose a person’s heart vascular disease using iPPG.

## Materials Availability

The database PURE is available in Ref. 14. Other data underlying the results presented in this paper are not publicly available at this time but may be obtained from the authors upon reasonable request.

## References

[1] Guyton C., Textbook of Medical Physiology, 11th ed., Elsevier Saunders (2006).

[2] Wu T. Blazek V. Schmitt H. J., “Photoplethysmography imaging: a new noninvasive and noncontact method for mapping of the dermal perfusion changes,” Proc. SPIE 4163, 62–70 (2000). PSISDG 0277-786X 10.1117/12.407646

[r3] Murakami K. Yoshioka M. Ozawa J., “Non-contact pulse transit time measurement using imaging camera, and its relation to blood pressure,” in 14th IAPR Int. Conf. Mach. Vis. Appl. (MVA), pp. 414–417 (2015). 10.1109/MVA.2015.7153099

[r4] Shao D. et al., “Non-contact monitoring breathing pattern, exhalation flow rate, and pulse transit time,” IEEE Trans. Biomed. Eng. 61(11), 2760–2767 (2014). IEBEAX 0018-9294 10.1109/TBME.2014.2327024 25330153

[r5] Kamshilin A. A. et al., “A new look at the essence of the imaging photoplethysmography,” Sci. Rep. 5(1), 10494 (2015). SRCEC3 2045-2322 10.1038/srep10494 25994481 PMC4440202

[r6] Sun Y. et al., “Noncontact imaging photoplethysmography to effectively access pulse rate variability,” J. Biomed. Opt. 18(6), 061205 (2012). JBOPFO 1083-3668 10.1117/1.JBO.18.6.061205 23111602

[r7] Kviesis-Kipge E. Rubins U., “Portable remote photoplethysmography device for monitoring of blood volume changes with high temporal resolution,” in 15th Biennial Baltic Electron. Conf. (BEC), pp. 55–58 (2016). 10.1109/BEC.2016.7743727

[r8] Lee K. Z. Hung P. C. Tsai L. W., “Contact-free heart rate measurement using a camera,” in Ninth Conf. Comput. and Rob. Vis., pp. 147–152 (2012). 10.1109/CRV.2012.27

[r9] Feng L. et al., “Dynamic ROI based on K-means for remote photoplethysmography,” in IEEE Int. Conf. Acoust., Speech and Signal Process. (ICASSP), pp. 1310–1314 (2015). 10.1109/ICASSP.2015.7178182

[r10] Trumpp A. et al., “Vasomotor assessment by camera-based photoplethysmography,” Curr. Dir. Biomed. Eng. 2(1), 199–202 (2016). 10.1515/cdbme-2016-0045

[r11] Humphreys K. Ward T. Markham C., “Noncontact simultaneous dual wavelength photoplethysmography: a further step toward noncontact pulse oximetry,” Rev. Sci. Instrum. 78(4), 044304 (2007). RSINAK 0034-6748 10.1063/1.2724789 17477684

[12] Blanik N. et al., “Hybrid optical imaging technology for long-term remote monitoring of skin perfusion and temperature behavior,” J. Biomed. Opt. 19(1), 016012 (2014). JBOPFO 1083-3668 10.1117/1.JBO.19.1.016012 24441875

[13] Holton B. D. et al., “Signal recovery in imaging photoplethysmography,” Physiol. Meas. 34(11), 1499–1511 (2013). PMEAE3 0967-3334 10.1088/0967-3334/34/11/1499 24149772

[r14] Stricker R. Müller S. Gross H., “Non-contact video-based pulse rate measurement on a mobile service robot,” in The 23rd IEEE Int. Symp. Rob. and Hum. Interactive Commun., pp. 1056–1062 (2014). 10.1109/ROMAN.2014.6926392

[r15] Hsu Y. Lin Y. L. Hsu W., “Learning-based heart rate detection from remote photoplethysmography features,” in IEEE Int. Conf. Acoust., Speech and Signal Process. (ICASSP), pp. 4433–4437 (2014). 10.1109/ICASSP.2014.6854440

[r16] Poh M. Z. McDuff D. J. Picard R. W., “Non-contact, automated cardiac pulse measurements using video imaging and blind source separation,” Opt. Express 18(10), 10762–10774 (2010). OPEXFF 1094-4087 10.1364/OE.18.010762 20588929

[17] Poh M. Z. McDuff D. J. Picard R. W., “Advancements in noncontact, multiparameter physiological measurements using a webcam,” IEEE Trans. Biomed. Eng. 58(1), 7–11 (2011). IEBEAX 0018-9294 10.1109/TBME.2010.2086456 20952328

[18] Butler M. J. et al., “Motion limitations of non-contact photoplethysmography due to the optical and topological properties of skin,” Physiol. Meas. 37(5), N27–N37 (2016). PMEAE3 0967-3334 10.1088/0967-3334/37/5/N27 27100666

[r19] Kumar M. Veeraraghavan A. Sabharwal A., “DistancePPG: Robust non-contact vital signs monitoring using a camera,” Biomed. Opt. Express 6(5), 1565–1588 (2015). BOEICL 2156-7085 10.1364/BOE.6.001565 26137365 PMC4467696

[r20] Lempe G. et al., “ROI selection for remote photoplethysmography,” in Bildverarbeitung für die Medizin, Meinzer H. P., et al., Eds., pp. 99–103, Springer, Berlin, Heidelberg (2013).

[r21] Bousefsaf F. Maaoui C. Pruski A., “Continuous wavelet filtering on webcam photoplethysmographic signals to remotely assess the instantaneous heart rate,” Biomed. Signal Process. Control 8(6), 568–574 (2013). 10.1016/j.bspc.2013.05.010

[r22] Bousefsaf F. Maaoui C. Pruski A., “Automatic selection of webcam photoplethysmographic pixels based on lightness criteria,” J. Med. Biol. Eng. 37(3), 374–385 (2017). IYSEAK 0021-3292 10.1007/s40846-017-0229-1

[r23] Bal U., “Non-contact estimation of heart rate and oxygen saturation using ambient light,” Biomed. Opt. Express 6(1), 86–97 (2014). BOEICL 2156-7085 10.1364/BOE.6.000086 25657877 PMC4317113

[r24] Huang R. Y. Dung L. R., “Measurement of heart rate variability using off-the-shelf smart phones,” Biomed. Eng. Online 15(1), 11 (2016). 10.1186/s12938-016-0127-8 26822804 PMC4731953

[r25] Woyczyk A. Fleischhauer A. V. Zaunseder S., “Skin segmentation using active contours and Gaussian mixture models for heart rate detection in videos,” in IEEE/CVF Conf. Comput. Vis. and Pattern Recognit. Workshops (CVPRW), pp. 1265–1273 (2020). 10.1109/CVPRW50498.2020.00164

[r26] Chong J. W. et al., “Non-contact HR monitoring via smartphone and webcam during different respiratory maneuvers and body movements,” IEEE J. Biomed. Health Inf. 25(2), 602–612 (2020). 10.1109/JBHI.2020.2998399 32750916

[r27] Tohma A. et al., “Evaluation of remote photoplethysmography measurement conditions toward telemedicine applications,” Sensors 21(24), 8357 (2021). SNSRES 0746-9462 10.3390/s21248357 34960451 PMC8704576

[r28] Po L. M. et al., “Block-based adaptive ROI for remote photoplethysmography,” Multimedia Tools Appl. 77(6), 6503–6529 (2018). 10.1007/s11042-017-4563-7

[29] Lian Y. et al., “Research on non-contact multi-person heart rate measurement method for intelligent education,” in 3rd Int. Conf. Inf. Sci., Parallel and Distributed Syst. (ISPDS), pp. 199–205 (2022). 10.1109/ISPDS56360.2022.9874225

[30] Ryu J. S. et al., “Research on the combination of color channels in heart rate measurement based on photoplethysmography imaging,” J. Biomed. Opt. 26(2), 025003 (2021). JBOPFO 1083-3668 10.1117/1.JBO.26.2.025003 33624458 PMC7901855

[31] Chopra K. et al., “A comprehensive examination of topographic thickness of skin in the human face,” Aesthet. Surg. J. 35(8), 1007–1013 (2015). 10.1093/asj/sjv079 26508650

[32] Kartynnik Y. et al., “Real-time facial surface geometry from monocular video on mobile GPUs,” (2019).

[33] Kovac J. Peer P. Solina F., “Human skin color clustering for face detection,” in The IEEE Region 8 EUROCON 2003. Comput. as a Tool, pp. 144–148 (2003). 10.1109/EURCON.2003.1248169

[34] Ryu J. S. et al., “A measurement of illumination variation-resistant noncontact heart rate based on the combination of singular spectrum analysis and sub-band method,” Comput. Methods Programs Biomed. 200, 105824 (2021). CMPBEK 0169-2607 10.1016/j.cmpb.2020.105824 33168271

[35] Alred G. J. Brusaw C. T. Oliu W. E., Handbook of Technical Writing, 7th ed., St. Martin’s, New York (2003).

[36] Qi H. et al., “Video-based human heart rate measurement using joint blind source separation,” Biomed. Signal Process. Control 31, 309–320 (2017). 10.1016/j.bspc.2016.08.020

[37] Cheng J. et al., “Illumination variation-resistant video-based heart rate measurement using joint blind source separation and ensemble empirical mode decomposition,” IEEE J. Biomed. Health Inf. 21(5), 1422–1433 (2017). 10.1109/JBHI.2016.2615472 27723609

[r38] Xu L. Cheng J. Chen X., “Illumination variation interference suppression in remote PPG using PLS and MEMD,” Electron. Lett. 53(4), 216–218 (2017). ELLEAK 0013-5194 10.1049/el.2016.3611

[39] Tarassenko L. et al., “Non-contact video-based vital sign monitoring using ambient light and auto-regressive models,” Physiol. Meas. 35, 807 (2014). PMEAE3 0967-3334 10.1088/0967-3334/35/5/807 24681430

